# High‐resolution expression profiling of selected gene sets during plant immune activation

**DOI:** 10.1111/pbi.13327

**Published:** 2020-01-27

**Authors:** Pingtao Ding, Bruno Pok Man Ngou, Oliver J. Furzer, Toshiyuki Sakai, Ram Krishna Shrestha, Dan MacLean, Jonathan D. G. Jones

**Affiliations:** ^1^ The Sainsbury Laboratory University of East Anglia Norwich Research Park Norwich UK; ^2^ Present address: The University of North Carolina Chapel Hill NC USA

**Keywords:** sequence capture, high‐resolution expression profiling, quantitative RNA‐seq, data visualization, transcriptional regulation, plant immunity, NLR

## Abstract

The plant immune system involves detection of pathogens via both cell‐surface and intracellular receptors. Both receptor classes can induce transcriptional reprogramming that elevates disease resistance. To assess differential gene expression during plant immunity, we developed and deployed quantitative sequence capture (CAP‐I). We designed and synthesized biotinylated single‐strand RNA bait libraries targeted to a subset of defense genes, and generated sequence capture data from 99 RNA‐seq libraries. We built a data processing pipeline to quantify the RNA‐CAP‐I‐seq data, and visualize differential gene expression. Sequence capture in combination with quantitative RNA‐seq enabled cost‐effective assessment of the expression profile of a specified subset of genes. Quantitative sequence capture is not limited to RNA‐seq or any specific organism and can potentially be incorporated into automated platforms for high‐throughput sequencing.

## Introduction

Sequence capture followed by next‐generation sequencing has broad applications in cost‐effective exploration of biological processes at high resolution (Jupe et al., 2013; Mercer et al., 2014). Genome‐wide RNA sequencing (RNA‐seq) over a time course can reveal the dynamics of differential gene expression. However, in many cases, only a limited set of genes are of interest and are repeatedly used as markers for certain biological processes. Sequence capture can help generate high‐resolution quantitative data sets to assess changes in abundance of selected genes. We previously used sequence capture to accelerate *Resistance* gene cloning (Jupe et al., 2013; Witek et al., 2016a,b), investigate immune receptor gene diversity (Van de Weyer et al., 2019) and investigate pathogen diversity and evolution (Jouet et al., 2019; Thilliez et al., 2019).

The plant immune system involves detection of pathogens via both cell‐surface and intracellular receptors. Both receptor classes can induce transcriptional reprogramming that elevates disease resistance (Jones and Dangl, 2006). To assess differential gene expression during plant immunity, we developed and deployed quantitative sequence capture (CAP‐I). We designed and synthesized biotinylated single‐strand RNA bait libraries targeted to a subset of defence genes and generated sequence capture data from 99 RNA‐seq libraries. We built a data processing pipeline to quantify the RNA‐CAP‐I‐seq data and visualize differential gene expression. Sequence capture in combination with quantitative RNA‐seq enabled cost‐effective assessment of the expression profile of a specified subset of genes. Quantitative sequence capture is not limited to RNA‐seq or any specific organism and can potentially be incorporated into automated platforms for high‐throughput sequencing.

## Materials and methods

### Plant material and growth condition

Mutants of *rrs1‐3 rrs1b‐1*, *eds1‐2*, *sid2‐2*, *sard1‐1 cbp60g‐1*, *myc2 myc3 myc4*, *tpr1 tpl tpr4* and *pad4‐1 ein2‐1 dde2‐2 sid2‐2* that were used in this study have been previously described (Saucet et al., 2015; Falk et al., 1999; Gallego‐Giraldo et al., 2011; Zhang et al., 2010; Fernández‐Calvo et al., 2011; Zhu et al., 2010; Tsuda et al., 2009). Seeds were sown on compost and plants were grown at 21°C with 10 h under light and 14 h in dark, and at 70% humidity.

### Bacterial infiltration assay and sample collection

All Pf0‐1 strains with different effectors were streaked from their glycerol stock in −70°C freezer on Petri dish plates with King’s B medium containing antibiotics for positive selection. Pf0‐1:AvrRps4 and Pf0‐1:AvrRps4^KRVY135‐138AAAA^ (Pf0‐1:AvrRps4^KRVYmut^) positive colonies were selected with 5 μg/ml tetracycline, 10 μg/ml chloramphenicol and 20 μg/ml gentamycin. Pf0‐1:AvrRpt2 were selected with 5 μg/ml tetracycline, 10 μg/ml chloramphenicol and 10 μg/ml kanamycin. Plates were growing in 28°C thermo incubator overnight. Fresh bacteria were streaked off from the plate surface with 1 ml clean pipette tips and resuspended in freshly prepared sterile 10 mM MgCl_2_ and spun with 2460 *g* for 3 min at room temperature. Discarded the supernatant and resuspended the pellet with 10 mM MgCl_2_. The concentration of bacteria was measured and indicated with the optical density at a wavelength of 600 nm (OD_600_). Final concentration of OD_600_ = 0.2 was used for infiltration with 1 ml needleless syringes. Two fully expanded leaves from a 5‐week‐old plant were infiltrated with one of the bacterial strains or just 10 mM MgCl_2_ resuspending buffer as mock. Six leaves from three plants were collected at 4 h post‐infiltration (hpi) for each genotype under one certain treatment. Leaves are snap‐frozen in liquid nitrogen for following up RNA extraction. Three batches of plants were grown under the same condition but on different dates, and samples collected from these three batches are used as three biological replicates.

### RNA extraction

All samples were kept in −70°C freezer before RNA isolation if the RNAs were not extracted immediately after sample collection (snapfrozen in liquid nitrogen). Total RNAs were extracted with Quick‐RNA™ Plant Miniprep Kit (Catalog No. R2024, Zymo Research, Irvine, CA, USA) following the protocol provided by Zymo Research. The quantities of RNAs were measured by Nanodrop and the qualities of RNAs were assessed with the RNA 6000 Nano Kit (Catalog No. 5067‐1511) on an Agilent 2100 Bioanalyzer System. mRNAs were purified with two times of enrichment using Dynabeads™ Oligo (dT)25 (Catalog No. 61002; Invitrogen^TM^, Carlsbad, CA, USA) from the total RNAs. The qualities and quantities of mRNAs were assessed with the RNA 6000 Pico Kit (Catalog No. 5067‐1513; Agilent, Santa Clara, CA, USA) on an Agilent 2100 Bioanalyzer System.

### cDNA library construction for RNA‐CAP‐I‐seq

mRNAs were submitted for first strand synthesis with Random Decamers (50 µM) (Catalog No. AM5722G; Invitrogen^TM^) and SuperScript™ IV Reverse Transcriptase kit (Catalog No. 18090200; Invitrogen^TM^). The second strand cDNA synthesis was carried out as previously described (Okayama and Berg, 1982; Rallapalli et al., 2014). Concentration of double strand cDNAs was quantified with the HS dsDNA Assay kit (Catalog No. Q32851; Invitrogen^TM^) on a Qubit Fluorometer. Illumina sequencing‐compatible cDNA libraries were constructed using tagmentation (Picelli et al., 2014). All libraries were barcoded with in‐house custom designed primers (Table S8) and assessed with the High Sensitivity DNA Kit (Catalog No. 5067‐4626; Aligent) on an Agilent 2100 Bioanalyzer System.

### CAP‐I bait design and RNA‐CAP‐I sequence capture

For enrichment of selected ERGs and controls, 2219 synthetic 120‐nt biotinylated RNA probes with 17 bp tiling were designed and synthesized, complementary to 52 gene regions (including promoter, coding, intron and terminators) totalling 261 616 bp from the reference genome of *Arabidopsis thaliana* Col‐0 (Swarbreck et al., 2008) (MYbaits; MYcroarray now is Arbor Biosciences, Ann Arbor, MI; https://arborbiosci.com/). Repetitive regions of total 18 800 bp within the targeted sequences were masked using RepeatMasker (Smit AFA, Hubley R & Green P. RepeatMasker Open‐4.0. 2013‐2015), and two highly represented baits with >10 MEGABLAST hits to the TAIR10 reference genome were removed (Altschul et al., 1990). All detailed information can also be found in our GitHub (Link). In preparation for sequencing, barcoded libraries were sized on the Agilent 2100 Bioanalyzer and then quantified using the Qubit Fluorometer and real‐time quantitative PCR (Catalog no. KK4824, Kapa Biosystems, Basel, Switzerland). Individual samples were pooled equimolarly. After multiplexing, the RNA‐CAP‐I library was carried out for sequence capture with CAP‐I baits following the protocol provided with blockers specifically for indices with 9 nucleotides. (https://arborbiosci.com/wp-content/uploads/2017/10/MYbaits-manual-v3.pdf)

### RNA‐CAP‐I‐seq on a NextSeq 500 sequencer

The multiplexed libraries were used as input following the NextSeq 500 instrument sample preparation protocol (Catalog no. 15048776, Illumina). With a recommended 1.8‐pM library concentration resulted in clustering density in our instrument (276 000 clusters/mm2). Samples were sequenced on a single flow cell of the NextSeq 500/550 High Output kit (75 cycles), using a 74‐cycle (single‐end) configuration. The sequencing run in the NextSeq 500 produced over 600 million single‐end reads with a Q30 ≥ 92.5%.

### Demultiplexing raw data from the NextSeq 500

Raw sequence data obtained from Illumina NextSeq500 sequencing platform are per‐cycle base call (BCL) format. As many analysis application tools require per‐read FASTQ format files as an input, we need to transform bcl file to fastq. A conversion software by Illumina called bcl2fastq version 2.20.0 (http://emea.support.illumina.com/downloads/bcl2fastq-conversion-software-v2-20.html) was used to demultiplex samples and convert the BCL format to FASTQ format. A sample sheet was prepared following the user guide (https://support.illumina.com/content/dam/illumina-support/documents/documentation/software_documentation/bcl2fastq/bcl2fastq2-v2-20-software-guide-15051736-03.pdf). The sample sheet contains sample identifier and a barcode or a barcode pair (nucleotide bases) and is provided to bcl2fastq for correct demultiplexing of the sample sequence reads. More detail about the command line usage of bcl2fastq tool can be obtained in the user guide. All raw reads post‐demultiplexing will be open access through the European Nucleotide Archive (ENA) under the accession number of PRJEB34520.

### Mapping reads to genome data, transcript annotation and profiling of gene expression

The single‐end reads for cDNA libraries were mapped to the *Arabidopsis thaliana* Col‐0 reference genome (TAIR10) using TopHat v.2.1.1 (Trapnell et al., 2012). Reads from the spike‐in genomic DNA were aligned to the reference genome using Bowtie2 v2.2.9 (Langmead and Salzberg, 2012). The resulting BAM files were sorted with SAMtools before downstream analysis (Li et al., 2009). With sorted BAM files, all downstream analysis following the pipeline of ‘atacR’ (Shrestha et al., 2018). All the data that we were not able to include in the supplemental materials are available in Github (https://github.com/slt666666/Ding_etal_2019_CAP_I ). All scripts and files we generated for this study are available in our Github (https://github.com/slt666666/Ding_etal_2019_CAP_I).

## Results and discussion

In previous work, we investigated changes in *Arabidopsis thaliana* defence gene expression in response to a bacterial effector after recognition via nucleotide‐binding leucine‐rich repeat intracellular immune receptors (NLRs). Specifically, we delivered the *Ralstonia solanacearum* effector PopP2 and studied responses to its recognition by the RPS4/RRS1‐R intracellular immune receptor complex (Sohn et al., 2014). We defined a subset of early response genes (ERGs) particularly responsive to NLR activation (Figure S1A; Tables S1 and S2). Expression of ERGs can be induced by both cell‐surface receptors and NLRs, but more rapidly and strongly induced when both classes of receptors are activated (Figure S1A). NLR‐dependent ERG up‐regulation was first observed at four hours post‐infiltration (4 hpi) (Figure S1B and C). To assess the roles of immune components during ERG activation, we measured ERG transcripts in selected immune‐deficient mutants compared to wild type (wt). Since these studies involved multiple replicates, mutant backgrounds and treatments, we applied complexity reduction via sequence capture to reduce sequencing costs.

**Figure 1:**
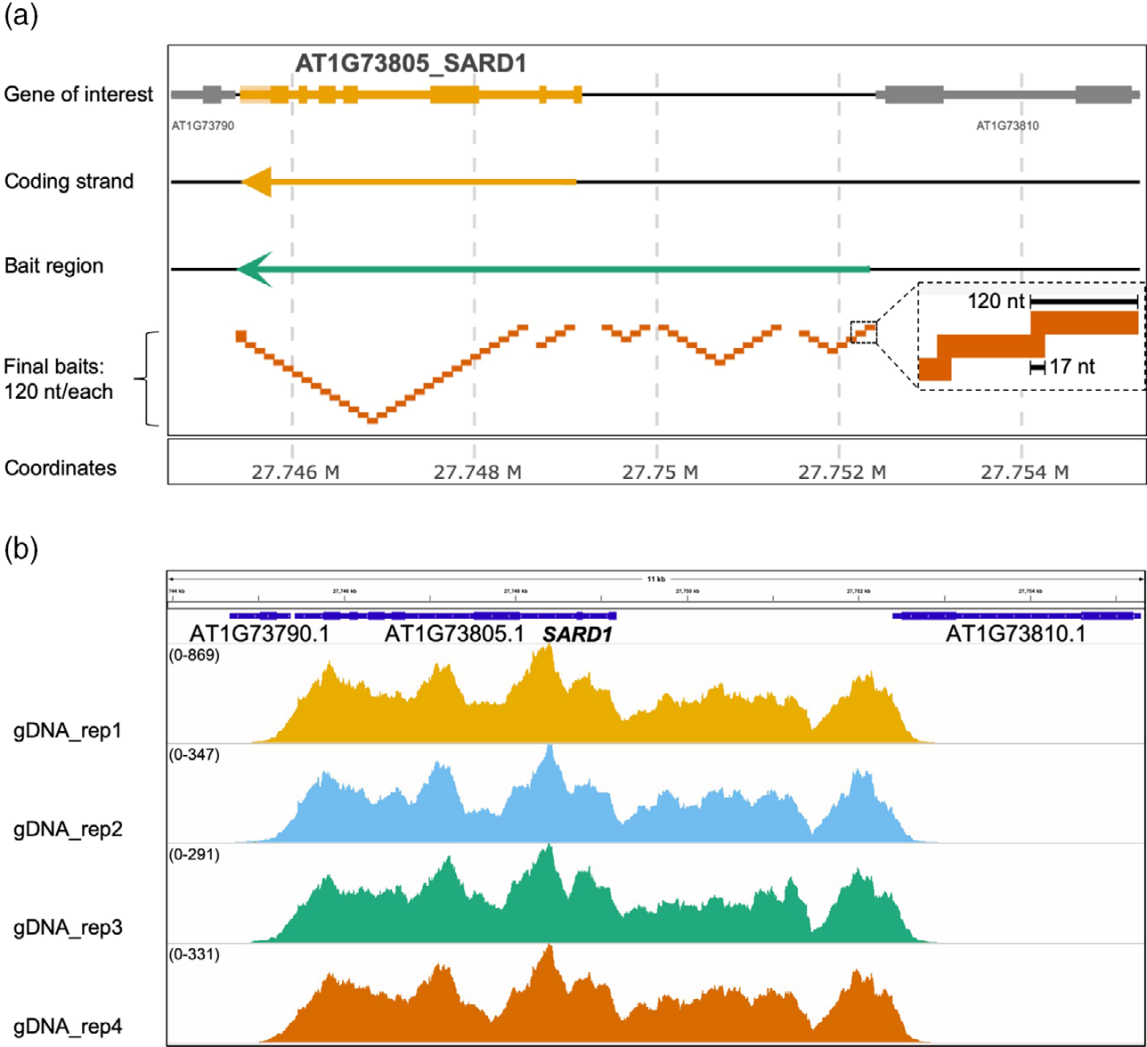
CAP‐I Bait Design and Validation. (a) Visualization of bait design on one of CAP‐I gene loci, *SARD1*. Using GFF file, here we present the genome organization of one CAP‐I gene locus, *SARD1*. Top row shows the annotated exons and introns and intragenic regions of CAP‐I gene locus and neighbouring gene loci. Second row shows the direction of the coding strand, here *SARD1* coding is on the reverse strand. The third row shows the orientation and the region that covers *SARD1* loci and putative promoter and terminator. The fourth strand shows the final non‐redundant baits we designed and how they are mapped to the CAP‐I target gene locus. The final baits are 120 nucleotides (nt) in length with 17 nt overlap for tilling. (b) Trial run of CAP‐I‐seq reads from genomic DNAs mapped to *SARD1* locus and visualized in a genome browser. Illumina sequencing reads of genomic DNA (gDNA) with four biological replicates in one CAP‐I capture shows 100% coverage on all CAP‐I gene loci including *SARD1*. See also Figure S1, Tables S1–S3.

We selected investigated 35 ERGs, and also 17 non‐ERGs as controls, based on their transcriptional regulation patterns (Figure S1A; Table S2) (Sohn et al., 2014). The ERGs include genes that are important for conferring full resistance to various plant pathogens and are involved in the biosynthesis of phytohormones, salicylic acid (SA) and pipecolic acid (Pip), including *ICS1*, *EDS5, PBS3, FMO1* and genes that encode the transcription factors (TFs) WRKY51 and SARD1 (Wildermuth et al., 2001; Nawrath et al., 2002; Nobuta et al., 2007; Zhang et al., 2010; Wang et al., 2011; Hartmann et al., 2018; Chen et al., 2018; Gao et al., 2011). Non‐ERG control genes include *UBQ10* and *ACT7*, as well as late immune response genes (Sohn et al., 2014), such as *PR1*, which is known to be activated by elevated SA (Cao et al., 1997). We included full‐length gene loci as templates for the capture bait design, spanning gene bodies (introns included) and putative promoters and terminators (Figure 1a). For promoters and terminators, we either defined them based on the intragenic sequence region between the coding sequence (CDS) of the target gene and the CDS of the immediate neighbouring genes (<4500 base pairs, or bps) or used 4500 bps upstream of the start codon or downstream of the stop codon as their promoters or terminators, respectively (Figure 1a). This was to minimize the loss of any important sequence information: some genes might need longer intragenic regions to be fully functional. All sequence templates were designed using the gene coding strand (Figure 1a).

After computationally extracting sequences from all 52 gene loci, we used our bait design pipeline to design a bait library (Figure 1a and S2A). We synthesized a set of 20 000 120‐mer single‐strand RNA probes (Figure 1a), which contains 2219 unique probes with 17‐nucleotide tiling and covering ~ 260 kb of the corresponding *Arabidopsis* genome regions (Figure S1A). We named this library as ‘Capture I’ (CAP‐I) for studies of plant innate immunity. To test the efficiency of CAP‐I for sequence capture, we performed one capture with libraries generated from *Arabidopsis* genomic DNA for NGS. We found all gene loci have 100% breadth of coverage (Figure 1b; Table S3), showing that CAP‐I enables capture of targeted sequences (Figure 1b). The pipeline generated one set of redundant baits in the region between two adjacent genes (Figure S2b), which could be condensed to provide additional capture capacity.

We then tested if CAP‐I can be used in RNA‐seq to assess quantitative changes in ERG transcripts. We used *Arabidopsis thaliana* accession Col‐0 as wt, and also investigated seven selected mutants in Col‐0 (Figure S2C). Resistance to *Ralstonia solanacearum* 1 (RRS1)‐S and RRS1B are NLRs of bacterial effector AvrRps4, and they function together with their paired NLRs resistant to *Pseudomonas (P.) syringae* 4 (RPS4) and RPS4B, respectively (Saucet et al., 2015); a *rrs1‐3 rrs1b‐1* mutant loses AvrRps4 responsiveness. EDS1 (the included mutant is *eds1‐2*) is required for immunity mediated by Toll/interleukin‐1 receptor/resistance (TIR)‐NLRs like RRS1 and RPS4 (Aarts et al., 1998). *SID2* (the included mutant is *sid2‐2*) encodes the enzyme ICS1, which is required for the biosynthesis of defence‐related phytohormone, SA (Dewdney et al., 2000; Wildermuth et al., 2001). SARD1 and its homolog Calmodulin‐binding protein 60‐like g (CBP60g) are master TFs required for transcriptional regulation of genes involved in pathogen‐associated molecular pattern (PAMP)‐triggered immunity (PTI), effector‐triggered immunity (ETI) and systemic acquired resistance (SAR) (Wang et al., 2009; Zhang et al., 2010; Wang et al., 2011; Sun et al., 2015; Ding and Redkar, 2018). MYC2 and its homologs MYC3 and MYC4 are basic helix‐loop‐helix TFs (the included mutant is *myc2 myc3 myc4*) required for jasmonic acid (JA)‐mediated resistance against bacteria (Fernández‐Calvo et al., 2011). TOPLESS (TPL) and its homologs TPL‐related 1 (TPR1) and TPR4 (the included mutant is *tpl tpr1 tpr4*) are putative transcriptional co‐repressors required for full resistance against the bacterium *P. syringae* pv. *tomato* DC3000 (hereafter DC3000) and DC3000 expressing AvrRps4 but not DC3000 expressing AvrRpt2, an effector recognized by RPS2, a non‐TIR‐NLR (Zhu et al., 2010)*.* Phytoalexin deficient 4 (PAD4), ethylene‐insensitive protein 2 (EIN2), delayed dehiscence 2 (DDE2, encoding an allene oxide synthase involved in jasmonic acid synthesis) and SID2/ICS1 (the included mutant is *pad4‐1 ein2‐1 dde2‐2 sid2‐2*) are proteins that are involved in different but interacting sectors in immune signalling networks (Tsuda et al., 2009).

Previously, we have defined the response induced by the bacterium *P. fluorescens* (Pf0‐1 EtHAn strain) carrying a mutant effector PopP2^C321A^ (Pf0‐1:PopP2^C321A^) as ‘PTI’ mediated by cell‐surface pathogen recognition receptors (PRRs) (Sohn et al., 2014). The Pf0‐1 strain carrying wt PopP2, recognized by RRS1‐R/RPS4, triggers an additional ETI response that we designate ‘PTI + ETI’. Here, we used Pf0‐1:AvrRps4 or Pf0‐1:AvrRpt2 to induce ‘PTI + ETI’. The responses induced by Pf0‐1:AvrRps4 or AvrRpt2 are named as ‘PTI plus TIR‐NLR‐mediated ETI’ (PTI + t‐ETI) and ‘PTI plus CC‐NLR‐mediated ETI’ (PTI + c‐ETI), respectively (Figure 3c). In addition, Pf0‐1 carrying the mutant effector AvrRps4^KRVY135‐138AAAA^ (Pf0‐1:AvrRps4^KRVYmut^) was included as ‘PTI’. We also included leaves infiltrated with buffer only, as a mock treatment, and no treatment on wt plants as an untreated control (Figure S2C). ERGs began to show significant up‐regulation in their transcripts at 4 hpi of Pf0‐1:PopP2 compared to Pf0‐1:PopP2^C321A^ (Sohn et al., 2014), so we collected our samples at 4 hpi for all treatments. For each combination of genotype and treatment, we collected 3 biological replicates; 99 samples in total (Figure S2C). We extracted RNAs from these samples and generated cDNA libraries. Each library was barcoded with custom index primers. In addition, we added genomic DNA libraries in the final multiplexed library as spike‐in controls for sequence capture. We applied one reaction of CAP‐I baits to capture the multiplexed libraries before sequencing.

**Figure 2:**
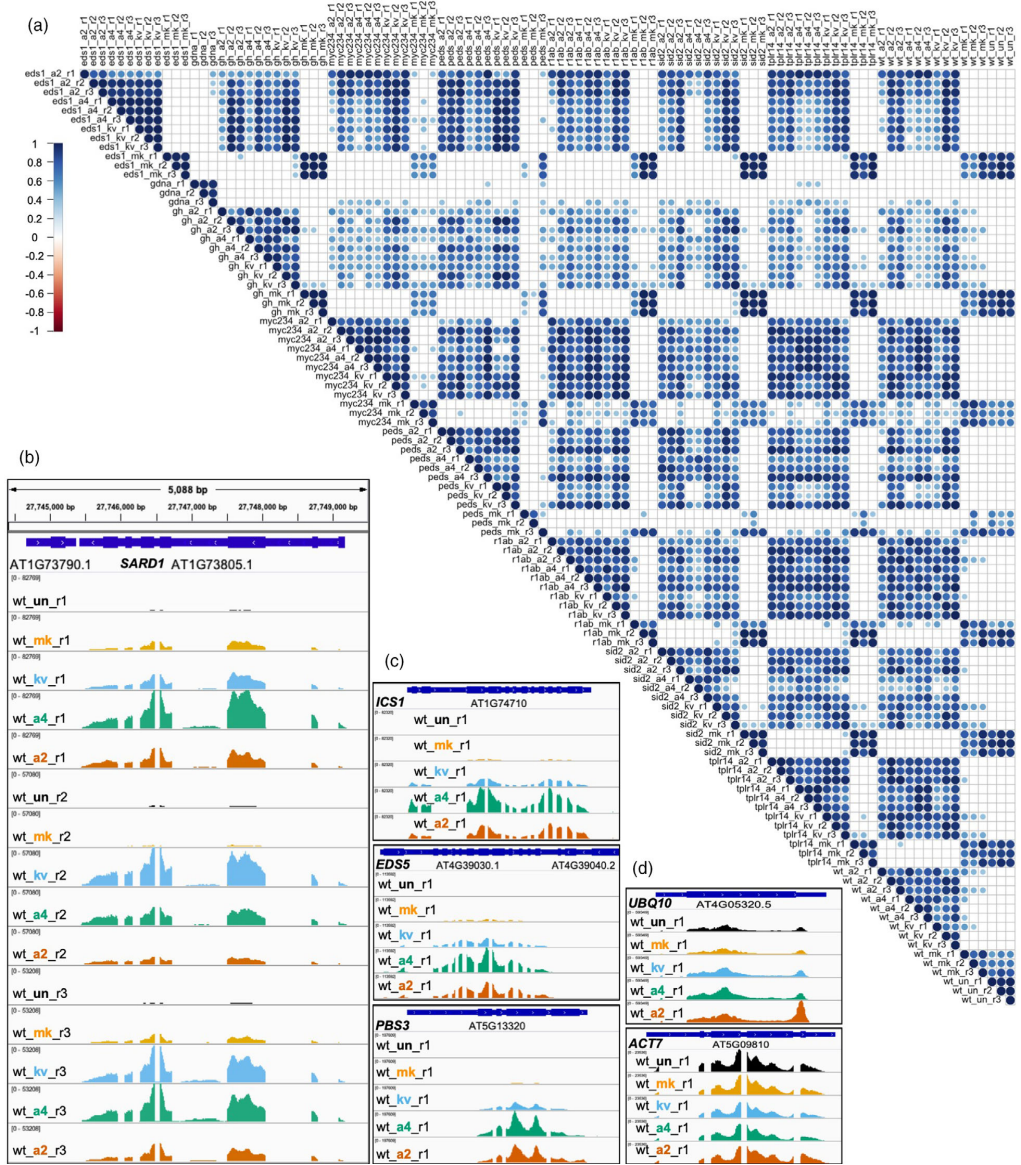
Reproducibility Test of RNA‐CAP‐I‐seq. (a) Correlation analysis of mapped reads from all individual libraries from RNA‐CAP‐I‐seq. All individual libraries including cDNA libraries and spike‐in gDNA libraries from the same CAP‐I‐seq are pairwisely compared. 1 indicates 100% positive correlation based on the distribution of reads, while ‐1 indicates 100% negative correlation. (b–d) Mapped reads before normalization are visualized in several CAP‐I gene loci in a genome browser. (b) Visualization of reads mapped to *SARD1* locus from wt samples. All three biological replicates (r1‐r3) of wt plants under five different treatments are visualized in IGV genome browser at *SARD1* locus. Black indicates untreated (un); orange indicates samples collected at 4 h post‐infiltration (hpi) of mock (10 mM MgCl_2_) treatment (mk); sky blue indicates samples collected at 4 hpi of Pf0‐1:AvrRps4^KRVYmut^ (kv); bluish green indicates samples collected at 4 hpi of Pf0‐1:AvrRps4 (a4); vermilion indicates samples collected at 4 hpi of Pf0‐1:AvrRpt2 (a2). See also Figure S2.

After demultiplexing, we retrieved single‐end reads for each individual library. We mapped the reads to CAP‐I target gene loci and assessed the mapping efficiency. We observed 100% breadth of coverage of full‐length transcripts for all gene loci except for *AT4G28410*, which encodes root system architecture 1 (RSA1). *RSA1* is specifically expressed in *Arabidopsis* root tissue, and all our samples are leaf tissues, so *RSA1* served as a good negative control for contamination introduced at any steps of library preparation and sequencing. Since no reads from 99 cDNA libraries of RNA‐CAP‐I‐seq mapped to the *RSA1* locus while 100% breadth of coverage in *RSA1* locus occurred in the gDNA spike‐in controls (Figure S3A), it demonstrates our baits are specific and sensitive to any changes in the quantity of targeted sequences. To test the reproducibility of each biological replicate, we generated a sample correlation plot (Figure 2a). Results of three biological replicates from the same combination of genotype and treatment group together based on their similarities, and the majority of the correlation coefficients between each pairwise comparison are above 0.8 (Figures 2a and S2D). Thus, the RNA‐CAP‐I‐seq method is highly repeatable. To check how well our RNA‐CAP‐I‐seq captured differential gene expression, we visualized the mapped reads in a genome browser. The overall expression pattern of *SARD1* gene in three biological replicates under all five different treatments is similar (Figure 2b). More reads were mapped to *SARD1* in the samples from ‘PTI’, ‘PTI + t‐ETI’ and ‘PTI + c‐ETI’ than those in mock or untreated samples, which is consistent with the previous observation of *SARD1* as one of the ERGs from the total RNA‐seq data (Sohn et al., 2014). Pathogen‐induced SA accumulation is required for plant immunity, and one major pathway of SA biosynthesis is *via* isochorismate (IC) (Dempsey et al., 2011). The IC pathway involves several enzymes that are required for the key catalytic steps, and encoded by *ICS1*, *EDS5* and *PBS3* (Rekhter et al., 2019; Torrens‐Spence et al., 2019). They are all ERGs and directly regulated by TFs SARD1 and CBP60g (Sohn et al., 2014; Sun et al., 2015). These three SA biosynthetic genes are usually transcriptionally co‐regulated in the activation of immunity and are also all highly induced in our ‘PTI’ and ‘PTI + ETI’ samples (Figure 2c). Furthermore, ‘PTI + ETI’ induces stronger expression of these genes than ‘PTI’ alone (Figure 2c), potentially through the regulation of SARD1 and CBP60g. In contrast, the transcripts of the house‐keeping genes, *UBQ10* and *ACT7,* are stable regardless of the treatments (Figure 2d).

**Figure 3:**
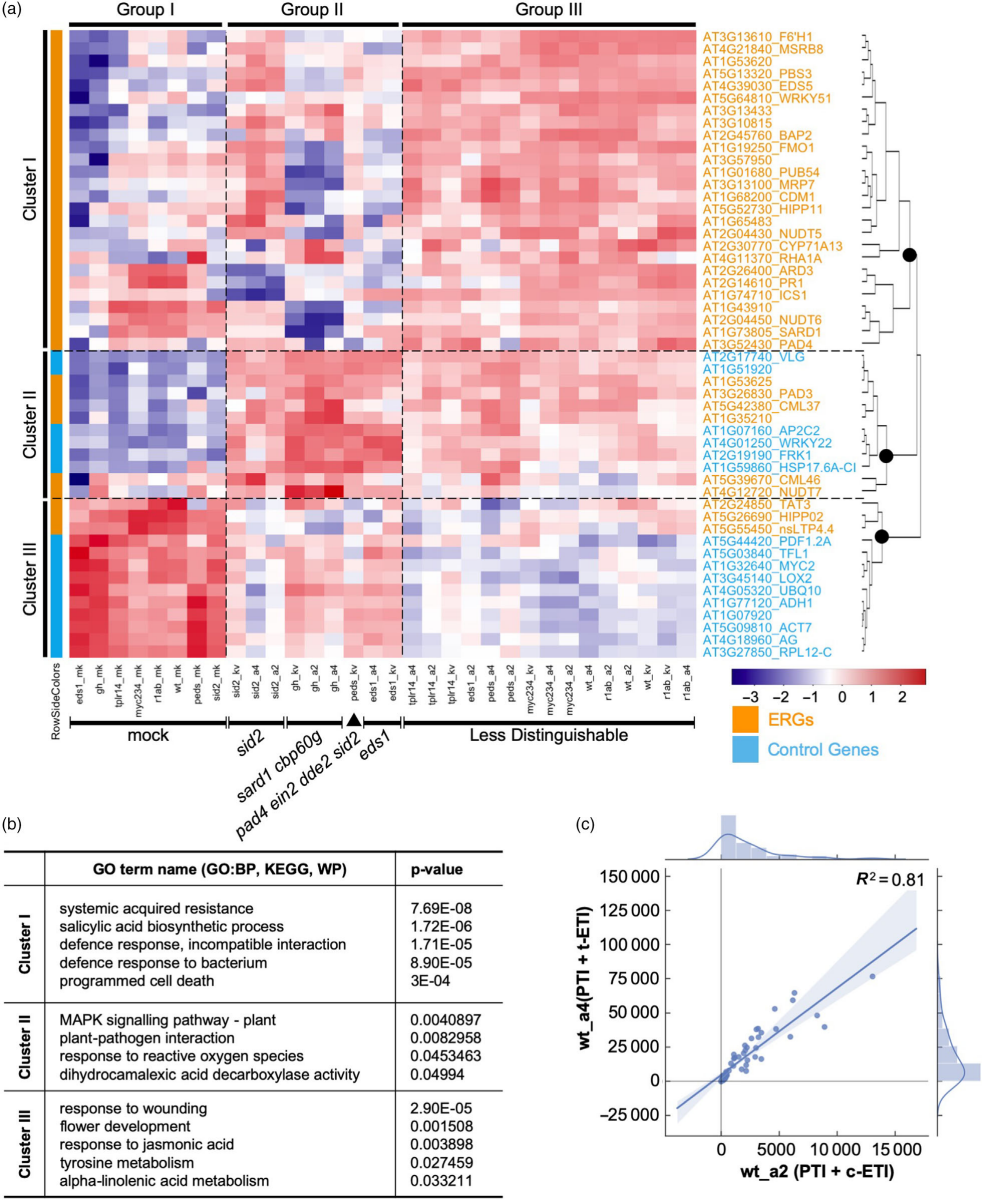
Quantification of RNA‐CAP‐I‐seq. (a) Cluster analysis of normalized read counts from each combination of conditions in comparison to untreated wt Col‐0 samples (wt_un). Each combination of conditions represents all combinations of each genotype (wt, eds1, r1ab, sid2, gh, myc234, tplr14, peds) with each treatment (mk, kv, a4, a2). CAP‐I genes form three major clusters based on their expression patterns cross all conditions. All conditions form three major groups based on their overall differential gene expression of CAP‐I genes. ERGs from CAP‐I are in orange and control genes are in sky blue. Heat map is based on mean z‐scores of three biological replicates. Redder colour indicates a higher value of z‐score, while bluer means a less value of z‐score. (b) Top hits of gene ontology (GO) terms based on their *p*‐values for CAP‐I genes in each cluster from (a). BP stands for biological process, and KEGG is based on the database from Kyoto Encyclopedia of Genes and Genomes. WP refers to WikiPathways database. (c) Comparison of differential gene expression patterns of all CAP‐I genes activated by ETI between RRS1/RPS4 and RPS2 in addition to PTI. See also Figure S3, Tables S4–S7.

**Figure 4:**
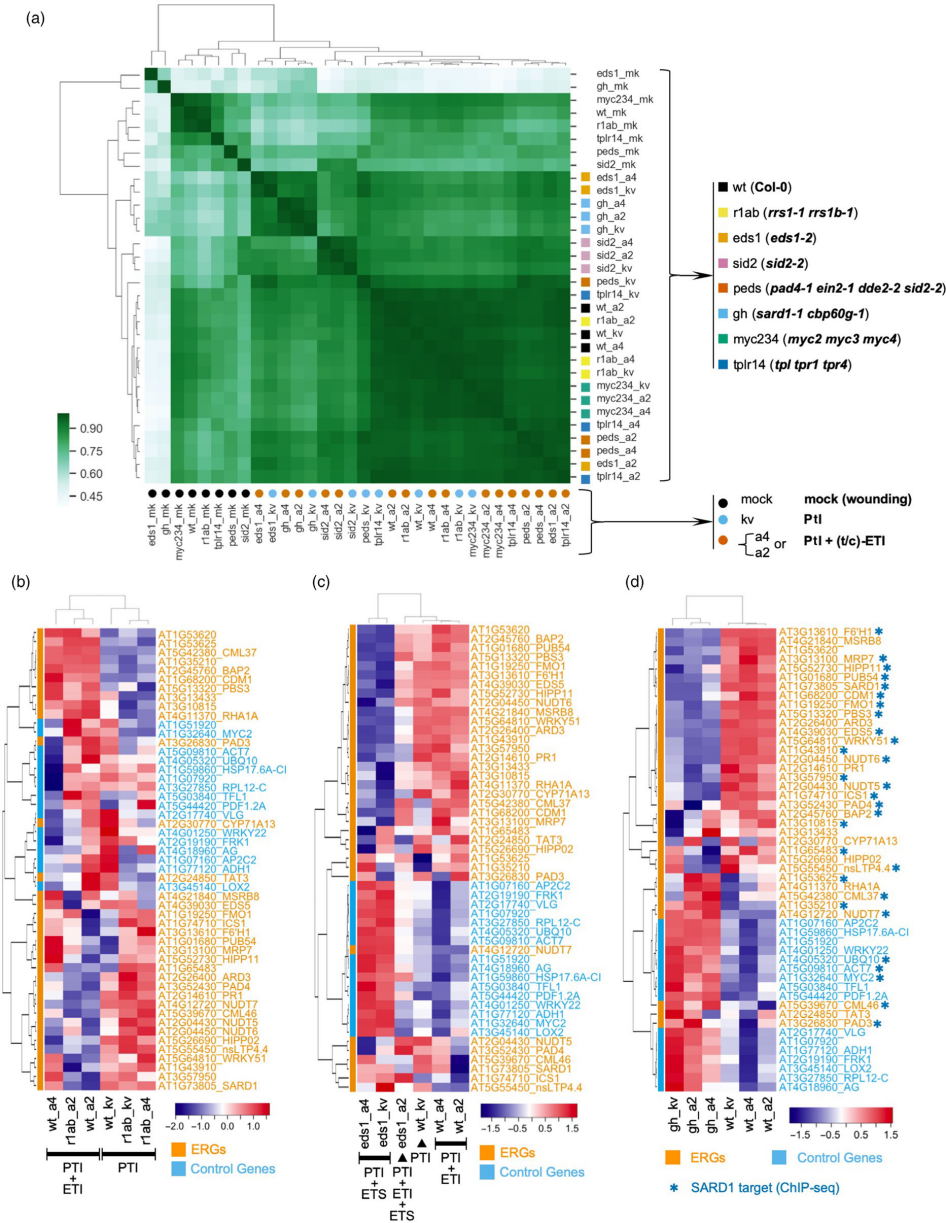
Correlation studies of RNA‐CAP‐I‐seq from different genotypes and treatments. (a) Correlation analysis with mapped and normalized reads from 32 different combinations of both genotypes and treatments. For treatments, we use colour‐filled circles to indicate, black circles stand for mock treatment. Sky blue circles are for Pf0‐1:AvrRps4^KRVYmut^ (kv). Vermilion circles are for Pf0‐1:AvrRps4 or AvrRpt2. For genotypes, we use colour‐filled squares to indicate, black squares are for wt Col‐0. Yellow squares are for *rrs1‐1 rrs1b‐1* double mutants. Orange squares are for *eds1‐2* (Col‐0) mutant. Reddish purple squares are for *sid2‐2* mutant, vermilion squares are for *pad4‐1 ein2‐1 dde2‐2 sid2‐2* quadruple mutants. Sky blue stands for *sard1‐1*. Bluish green stands for *myc2/3/4, blue* is for TOPLESS mutants *tprl tpr1 trpr4*. (b–d) differential gene expression are visualized with heat maps. (b) Heat map of differential expression of CAP‐I genes in *rrs1 rrs1b* double mutants compared to wt. (c) Heat map of CAP‐I genes in *eds1* mutant compared to wt. (d) Heat map of CAP‐I genes in sard1 cbp60g mutants compared to wt. See also Figure S4.

Though we observed what we expected from the mapped reads, they required normalization for statistical analysis of relative gene expression. For this, we have developed an R package to normalize and visualize the data generated with sequence capture (Shrestha et al., 2018). From the parameter of ‘Goodness Of Fit’, we found that not all selected control genes are suitable for normalization as some of them are highly variable across 99 samples (Figure S3B). After normalization, we obtained a balanced read distribution with low variation across all samples (Tables S4 and S5), enabling statistical analysis for differential gene expression. In the clustering analysis, we retrieved three main clusters of genes based on their expression patterns in all 32 different treatments compared to untreated Col‐0 samples (Figure 3a; Table S6). The majority of ERGs are in Cluster I and mostly are immunity related, while Cluster III comprises predominantly control genes (Figure 3b; Table S7). Cluster II contains equal numbers of ERGs and control genes (Figure 3a and b). From the same analysis, we also identified three groups of conditions categorizing combinations of genotypes and treatments. Regardless of the genotype, all mock treated samples are clustered in Group I with similar expression patterns of CAP‐I genes, indicating they serve as a good negative control for other treatments. In Group III, overall expression of CAP‐I genes had no discernable pattern compared to that in Groups I and II. In Group II, we were able to identify mutants that have greater impacts on ERG expression pattern in response to treatments (Figure 3a). All Pf0‐1‐treated samples in *sid2* mutant exhibit similar expression profiles, as do those in *sard1 cbp60g* double mutant. These indicate that ICS1 or SARD1/CBP60g is required for the activation of both ‘PTI’ and ‘PTI + ETI’. Consistent with EDS1 being required for AvrRps4‐ but not AvrRpt2‐induced ETI, our results also show that ERGs in *eds1* are induced less by Pf0‐1:AvrRps4 and Pf0‐1:AvrRps4^KRVYmut^ (eds1_a4 and eds1_kv) in comparison to those induced by Pf0‐1:AvrRpt2 (eds1_a2) (Figure 3a). We also observed that ERGs are induced less in a *pad4 ein2 dde2 sid2* quadruple mutant (*peds*) than in wt by ‘PTI’, which is consistent with previous reports (Tsuda et al., 2009; Hillmer et al., 2017). However, we did not see a strong ERG difference between *peds* and wt in response to ‘PTI + ETI’ (Figure 3a).

t‐ETI and c‐ETI confer resistance *via* different types of NLRs and signalling components (Aarts et al., 1998; Jones and Dangl, 2006). However, there is no previously reported side‐by‐side comparison of TIR‐NLR‐ and CC‐NLR‐induced genes upon NLR activation. Here, we compared the induction patterns of ERGs in wt treated with ‘PTI + t‐ETI’ and ‘PTI + c‐ETI’, and they significantly resemble each other for all CAP‐I genes (*R* ^2^ = 0.81) (Figure 3c). As the 32 conditions are combinations of both genotypes and treatments, we checked the correlation of gene expression patterns with either genotypes or treatments separately (Figure 4a). Gene expression patterns from the treatments of ‘PTI + t‐ETI’ and ‘PTI + c‐ETI’ within the same genotype tend to group together, rather than with ‘PTI’ (Figure 4a), which further proves that gene expression patterns induced by TIR‐NLRs and CC‐NLRs at early immune activation stages are similar.

We examined differential gene expression between each individual mutant and wt. As expected, in both *eds1* and *rrs1 rrs1b* mutants, gene expression patterns are similar between the two treatments of Pf0‐1:AvrRps4 and Pf0‐1:AvrRps4^KRVYmut^, because both EDS1 and RRS1/RRS1B are required for AvrRps4‐induced ETI. Loss‐of‐function of the AvrRps4 receptors (*rrs1 rrs1b*) or the downstream signalling component EDS1 (*eds1*) resembles the loss‐of‐recognition of AvrRps4 due to the mutation of AvrRps4 (Pf0‐1:AvrRps4^KRVYmut^) in wt plants (Figure 4b and c). On the other hand, EDS1 and RRS1/RRS1B are not required for AvrRpt2 recognition, so Pf0‐1:AvrRpt2 can still induce both PTI and ETI in *eds1* and *rrs1 rrs1b* mutants (Figure 4b and c).

The TFs SARD1 and CBP60g bind to the promoters of defence genes to regulate their expression (Zhang et al., 2010; Sun et al., 2015). We observed that most ERGs that are down‐regulated in *sard1 cbp60g* mutants are also identified as targets of SARD1 from chromatin immunoprecipitation followed by sequencing (ChIP‐seq) of SARD1 (Figure 4d) (Sun et al., 2015).

The *sid2* mutant is known to have no expression of the *ICS1* gene and compromised SA accumulation induced by pathogens, so we expected to see that SA‐induced genes were also down‐regulated. We observed that genes induced by SA and up‐regulated during SAR, specifically *PR1 and Acireductone Dioxygenase 3* (*ARD3*), were both down‐regulated in *sid2* (Figure S4A). *SARD1* is also down‐regulated in *sid2*, indicating that SARD1‐dependent regulation of *ICS1* and SA biosynthesis can in turn positively regulate *SARD1* gene expression. TF WRKY51 and its homolog WRKY50 positively regulate SA signalling and negatively regulate JA signalling (Gao et al., 2011). In *wrky50 wrky51* loss‐of‐function mutants, *Plant Defensin 1.2A* (*PDF1.2A*) is down‐regulated in response to JA (Gao et al., 2011). Here, we found in a *sid2* mutant, *WRKY51* is down‐regulated, while *PDF1.2A* is up‐regulated (Figure S4A), which is consistent with the negative expression association between *WRKY51* and *PDF1.2A*. In addition, we found *Cytochrome P450 Monooxygenase 71A13* (*CYP71A13*) is down‐regulated in *sid2* upon activation of innate immunity, indicating that SA might play positive regulatory roles in camalexin synthesis (Nafisi et al., 2007).

The expression of JA response genes *Tyrosine Aminotransferase 3* (*TAT3*) and *Lipoxygenase 2* (*LOX2*) but not *PDF1.2A* is positively regulated by MYC2 and its homologues MYC3 and MYC4 (Fernández‐Calvo et al., 2011; Goossens et al., 2015). In our RNA‐CAP‐I‐seq data, we found *MYC2, TAT3* and *LOX2* are down‐regulated in *myc2 myc3 myc4* triple mutants, whereas *PDF1.2A* is up‐regulated in the triple mutant in response to activation of innate immunity (Figure S4B).

TOPLESS mutants *tpr1 tpl tpr4* show enhanced susceptibility to bacteria DC3000 and DC3000 carrying AvrRps4 (Zhu et al., 2010). However, this cannot be simply explained by the expression pattern of ERGs, as we found no clear reduction of ERGs in *tpr1 tpl tpr4* mutants (Figure S5C). Previously, TOPLESS proteins were reported as transcriptional co‐repressors, but there is only slight evidence in our data of TOPLESS repressor activity towards a few specific genes. Here, we found some defence‐related ERGs are down‐regulated, while others are up‐regulated, in response to both ‘PTI + t‐ETI’ and ‘PTI + c‐ETI’ compared to ‘PTI’, which indicates that TOPLESS proteins may play dual functions or indirect roles in regulating ERGs. As there is no ChIP‐seq data of TOPLESS proteins or related histone modification marks available, their functions remain unclear. Our data, together with previous reports, nevertheless indicate a complex contribution of TOPLESS proteins in regulating genes induced during plant immunity (Figure S4C) (Zhu et al., 2010).

The *peds* mutant carries mutations in genes from four major immune sectors: PAD4 (*pad4*), ethylene (*ein2*), JA (*dde2*) and SA (*sid2*) (Tsuda et al., 2009). We observed that PAD4, SA and JA response genes are down‐regulated in *peds*, including *PAD4, ICS1*, *EDS5*, *WRKY51*, *CYP71A13*, *MYC2*, *TAT3* and *LOX2* (Figure S4D). It has been reported that the *PEDS‐*represented phytohormone network is required for achieving higher amplitude of transcriptional reprogramming during early CC‐NLR‐activated ETI in addition to PTI than during PTI alone (Mine et al., 2018). However in that report (Mine et al., 2018), the authors used DC3000 instead of Pf0‐1 in our case, which can not only trigger ‘PTI + ETI’ but the background effectors in DC3000 can also trigger effector‐triggered susceptibility (‘ETS’), so our results using Pf0‐1 are ‘cleaner’. We showed a greater expression difference of ERGs activated by ‘PTI’ and by ‘PTI + ETI’ in *peds* mutant compared to wt (Figure S4D). Like AvrRpt2, AvrRpm1 is also recognized by a CC‐NLR, resistance to *P. syringae* pv *maculicola* 1 (RPM1) and activates ETI (Innes et al., 1993; Jones and Dangl, 2006). Unlike AvrRpt2‐induced ETI, AvrRpm1‐induced ETI does not require *PEDS‐*represented phytohormone network to achieve a high‐amplitude transcriptional reprogramme within the early time window of ETI activation (Mine et al., 2018). Data from the same report indicate that *RPS2*, but not *RPM1*, gene expression is highly reduced in *peds* when ETI was activated (Mine et al., 2018). From this, we hypothesize that *RPS2* gene expression might be regulated through these four sectors, explaining why all AvrRpt2‐induced ERGs are delayed in contrast to AvrRpm1‐induced ETI.

Here, using a limited subset of genes (CAP‐I), we could distinguish gene expression profiles during ‘PTI’, ‘PTI + c‐ETI’, ‘PTI + t‐ETI’ in various mutants, particularly the immune gene regulatory components EDS1, ICS1 and SARD1/CBP60g. Inclusion of additional innate immunity genes in the bait library should enable us to distinguish mutants with enhanced resolution. In addition, as all steps for CAP‐I are easy to follow and reproducible, CAP‐seq can be further implemented in an automated platform for more high‐throughput applications.

Single cell RNA‐seq (scRNA‐seq) for signature genes is available for some plant tissues (Ryu et al., 2019; Shulse et al., 2019) and could be combined with capture‐seq. A set of 100 marker genes has been defined for *Arabidopsis* that can be used to predict the total transcriptome for each species (Biswas et al., 2017); these could be incorporated into future capture‐seq bait library design. Capture‐seq is also capable of comparing the changes in the abundance of any DNA sequences, so it is not limited to cDNA libraries, but can be used in other types of DNA libraries, such as ChIP‐seq and Assay for Transposase‐Accessible Chromatin using sequencing (ATAC‐seq) (Park, 2009; Buenrostro et al., 2015). For one single experiment of sequence capture with CAP‐I baits in this study, it was estimated to be three times cheaper than using the conventional genome‐wide RNA‐seq, including consumables from synthesizing baits, library preparation and sequencing. Furthermore, the synthesized CAP‐I baits can be used for at least a hundred more sequence capture reactions that are similar to this study. In addition, ten times more multiplexed libraries than this study can be included in the same flow cell to achieve the same read depth and coverage compared to the conventional genome‐wide RNA‐seq for differential gene expression analysis. Finally, capture‐seq could also be used to investigate expression of specific pathogen genes during host colonization (Pathogen Enrichment Sequencing: PenSeq) (Jouet et al., 2019; Thilliez et al., 2019). In summary, sequence capture provides an extremely versatile and cost‐effective method to investigate changes in expression of any designated gene set.

## Conflict of interest

The authors declare no competing interests.

## Author contributions

PD and JDGJ conceptualized the study. Experiments and methods of RNA‐CAP‐I‐seq were designed by PD and were carried out by PD and BN. CAP‐I baits and barcodes are designed by PD and OJF. Data analysis was performed by PD, DM, RKS and TS. The original draft was written by PD, and reviewed and edited by JDGJ, BN, OJF, TS, RKS and DM.

## Supporting information


**Figure S1** Time‐series expression of CAP‐I genes under different conditions of immune activation.

**Figure S2** CAP‐I bait design and RNA‐CAP‐I‐seq experimental design.

**Figure S3** Overall quality assessment of RNA‐CAP‐I‐seq data.

**Figure S4** Heat maps of differential gene expression of mutants individually compared to wt.

**Table S1** Information_of_ERGs_and_Control_Genes_in_CAP‐I.

**Table S2** Time‐series_Expression_of_CAP‐I_genes.

**Table S3** Coverage_Information_of_CAP‐I_gDNA_seq_Trial.

**Table S4** Read_Counts_of_RNA‐CAP‐I‐seq_before_Normalisation.

**Table S5** Read_Counts_of_RNA‐CAP‐I‐seq_post_Normalisation.

**Table S6** Log_Matrix_of_RNA‐CAP‐I‐seq_Normalised_to_wt_un.

**Table S7** Geno_Ontology_Information_for_Clusters_in_Differential_Gene_Expression_Heatmap.

**Table S8** Barcode_Information_for_RNA‐Cap‐I_Seq.
