# Supplementary figures and images for: High‐resolution expression profiling of selected gene sets during plant immune activation
Source: Plant Biotechnol J. 2020 Jan 27;18(7):1610–9. doi: 10.1111/pbi.13327 (PMC7292544; doi:10.1111/pbi.13327)

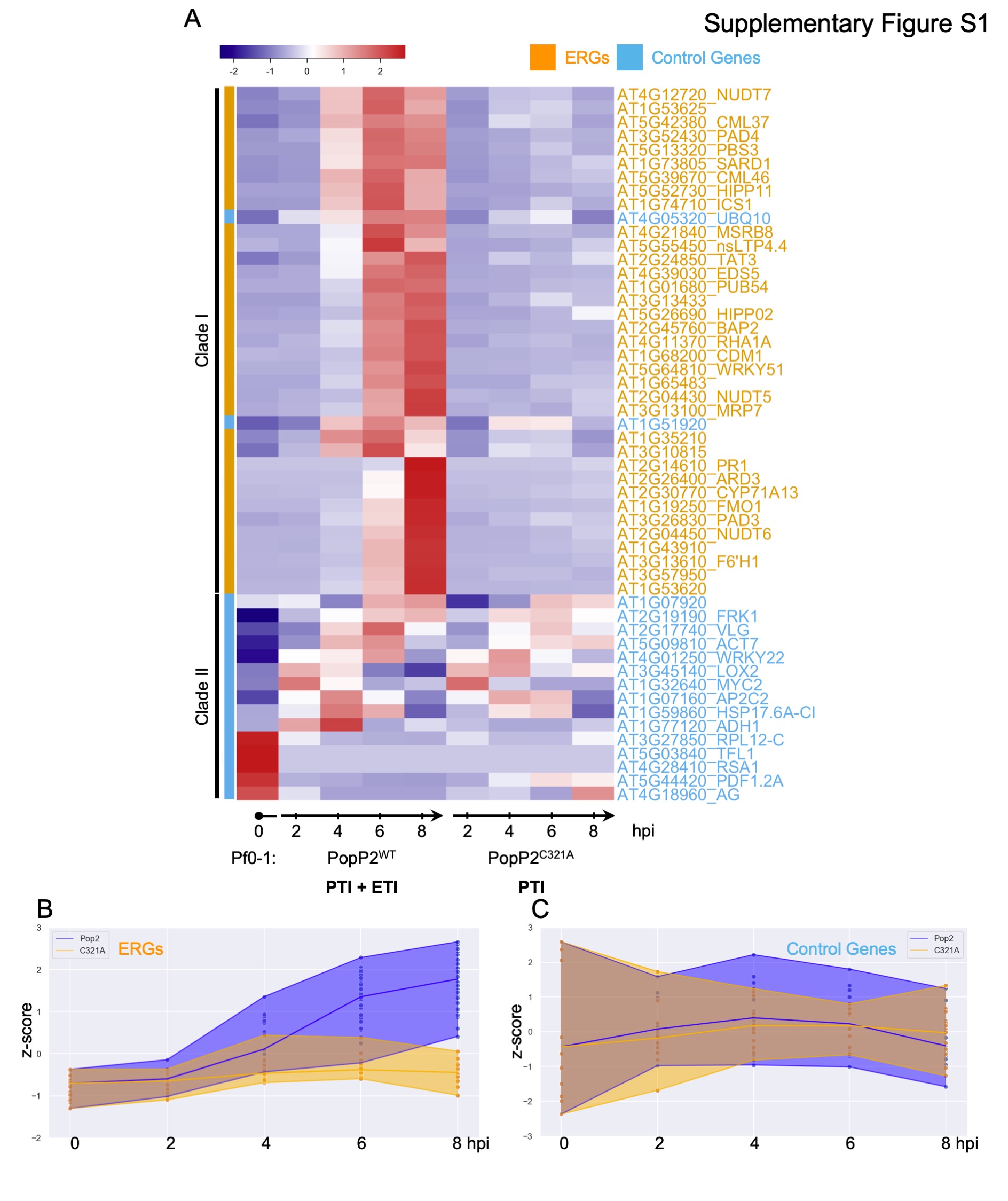

Supplement: Supplementary file 1 — Figure S1 Time‐series expression of CAP‐I genes under different conditions of immune activation. [file PBI-18-1610-s012.jpg]

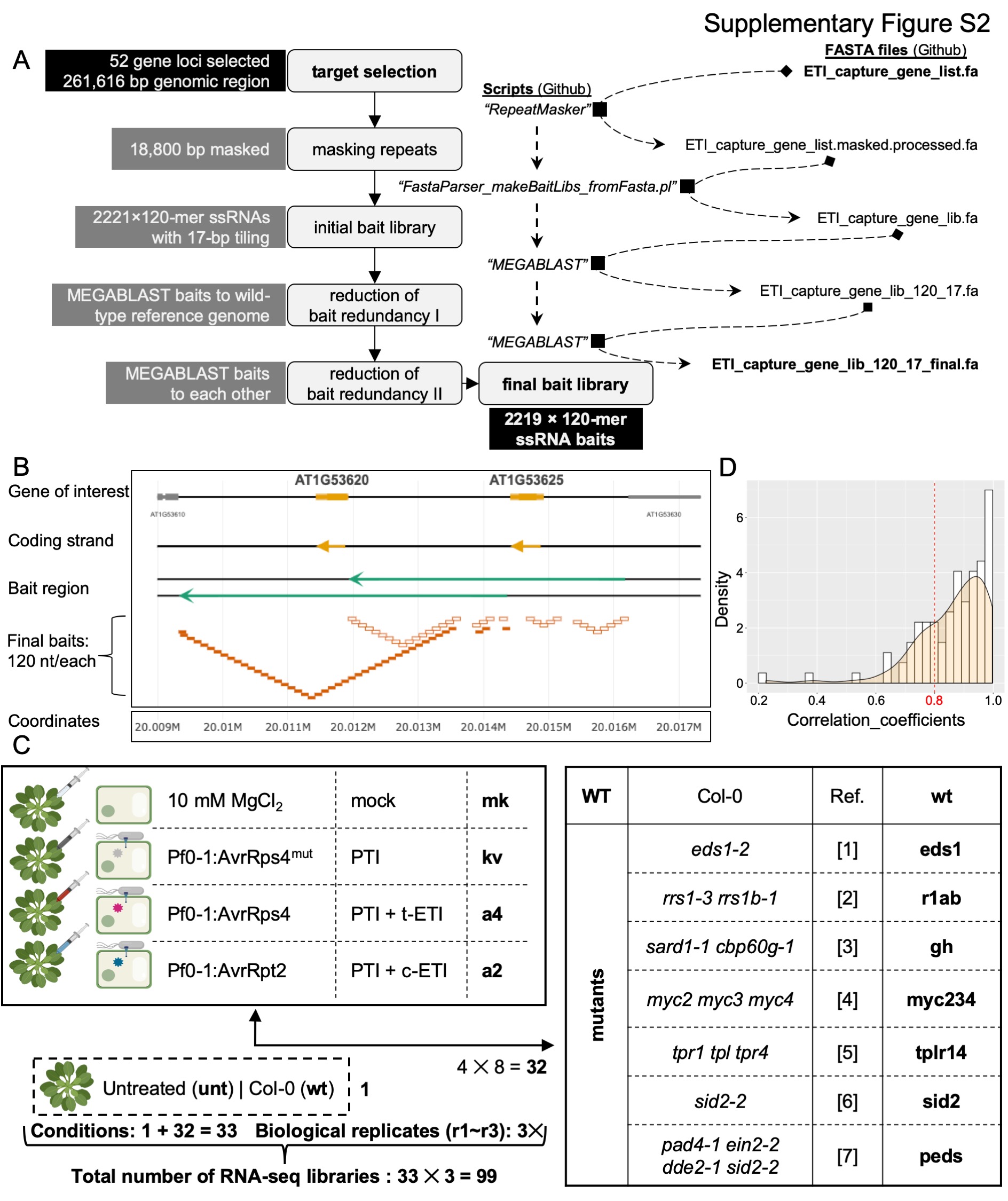

Supplement: Supplementary file 2 — Figure S2 CAP‐I bait design and RNA‐CAP‐I‐seq experimental design. [file PBI-18-1610-s011.jpg]

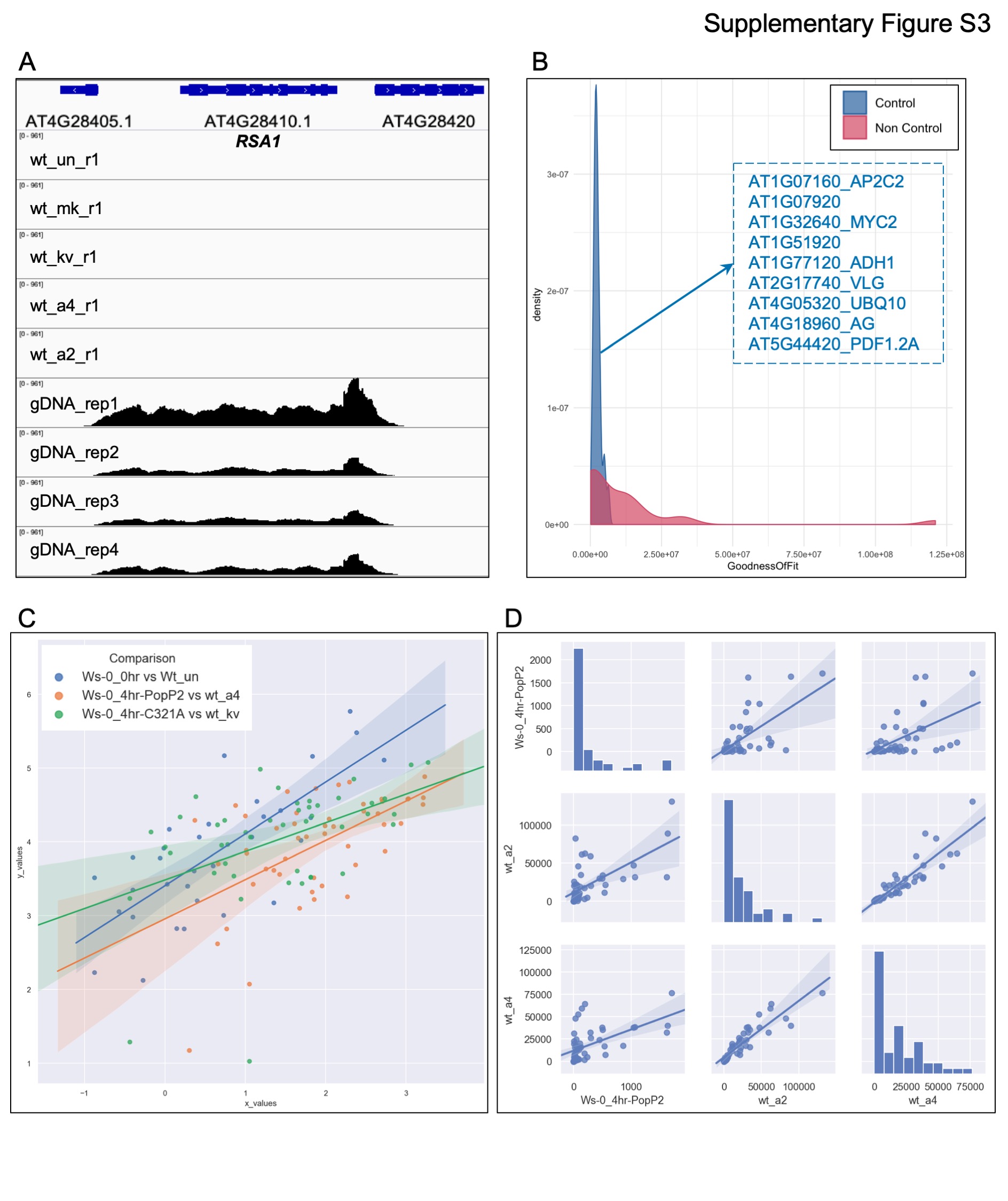

Supplement: Supplementary file 3 — Figure S3 Overall quality assessment of RNA‐CAP‐I‐seq data. [file PBI-18-1610-s010.jpg]

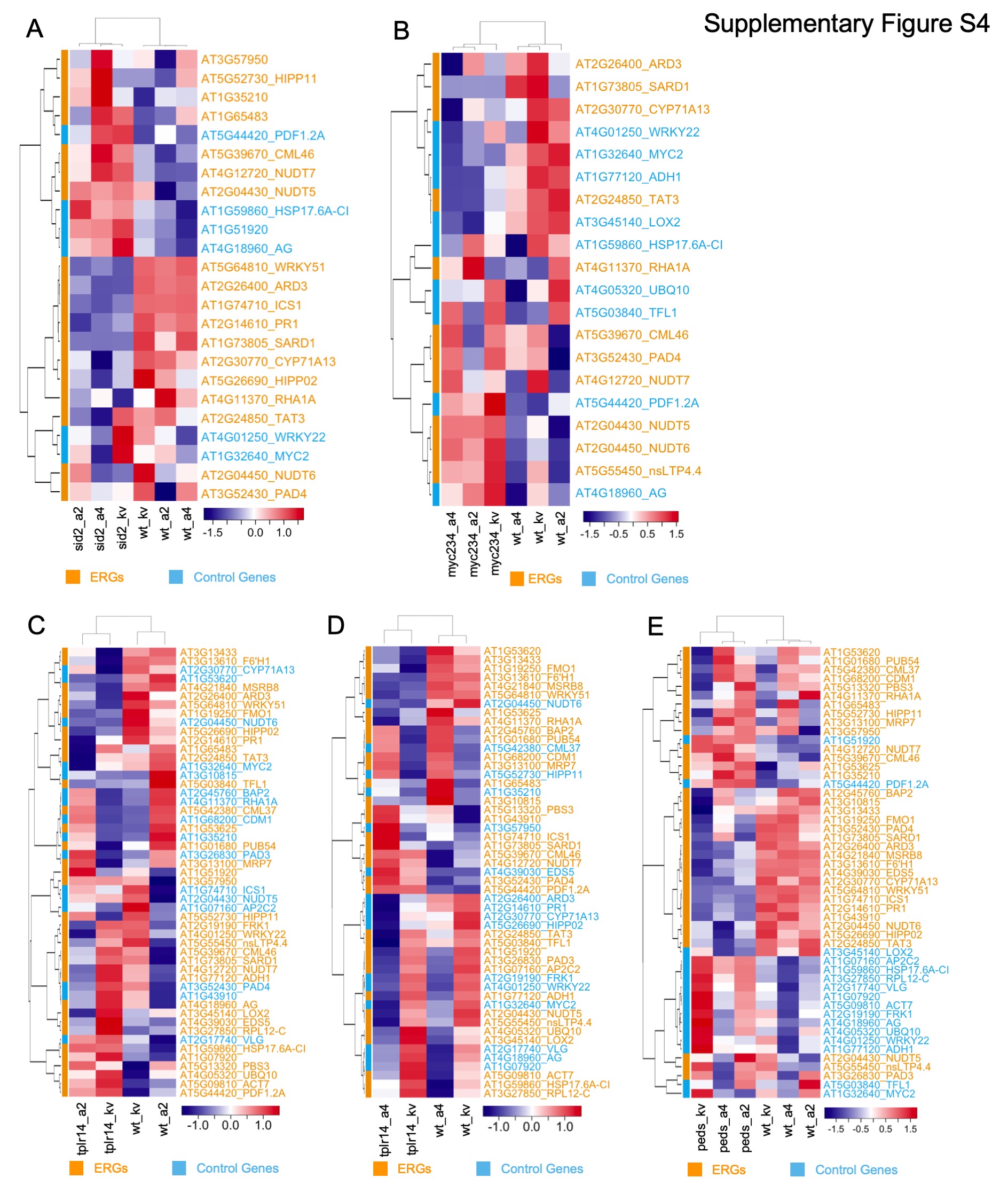

Supplement: Supplementary file 4 — Figure S4 Heat maps of differential gene expression of mutants individually compared to wt. [file PBI-18-1610-s009.jpg]
